# Di-μ-carbonyl-bis­[bis­(triphenyl­phos­phane)rhodium(0)](*Rh*—*Rh*) acetone disolvate^1^

**DOI:** 10.1107/S1600536812043528

**Published:** 2012-10-27

**Authors:** Petia G. Gueorguieva, Scott A. Laneman, George G. Stanley, Frank R. Fronczek, Steven F. Watkins

**Affiliations:** a Department of Chemistry, Louisiana State University, Baton Rouge, LA 70803-1804, USA

## Abstract

The dirhodium complex, [Rh_2_(C_18_H_15_P)_4_(CO)_2_]·2(CH_3_)_2_CO, has crystallographic twofold symmetry and the Rh—Rh distance is 2.6266 (8) Å. The four atoms proximate to each Rh atom [Rh—P = 2.3222 (7) and 2.3283 (8) Å, and Rh—C = 1.961 (3) and 2.045 (3) Å] form a distorted tetra­hedron with large deviations from the putative tetra­hedral angles [r.m.s. deviation = 23 (1)°]. The six angles more closely approximate those of a trigonal bipyramid [r.m.s. deviation = 14 (1)°] with one missing equatorial ligand. The two bridging carbonyl ligands are much more linearly coordinated to one Rh [Rh—C O = 151.0 (2)°] than to the other [127.0 (2)°], and the two Rh_2_CO planes form a dihedral angle of 45.43 (5)°. The two acetone solvent mol­ecules are disordered, and their estimated scattering contribution was subtracted from the observed diffraction data using the SQUEEZE routine in *PLATON* [Spek (2009 ▶). *Acta Cryst.* **D65**, 148–155].

## Related literature

For other dirhodium complex structures, see CCDC Refcode QAFHEM: Dzik *et al.* (2010 ▶), YOSMEZ: Okazaki *et al.* (2009 ▶), DEFJII: Douglas *et al.* (2005 ▶), TPCDRH10: Singh *et al.* (1973 ▶). For a description of the Cambridge Structural Database, see: Allen (2002 ▶). For the use of SQUEEZE, see: Spek (2009 ▶).
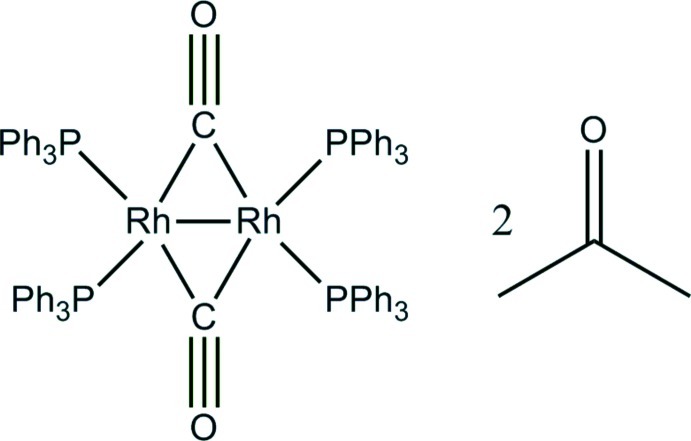


## Experimental

### 

#### Crystal data

[Rh_2_(C_18_H_15_P)_4_(CO)_2_]·2C_3_H_6_O
*M* *_r_* = 1427.16
Monoclinic, 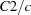

*a* = 23.535 (3) Å
*b* = 13.0758 (11) Å
*c* = 24.650 (2) Å
β = 115.67 (2)°
*V* = 6837.1 (16) Å^3^
*Z* = 4
Mo *K*α radiation
μ = 0.63 mm^−1^
*T* = 298 K
0.38 × 0.38 × 0.23 mm

#### Data collection

Enraf–Nonius CAD-4 diffractometer
Absorption correction: ψ scan (North *et al.*, 1968 ▶) *T*_min_ = 0.797, *T*_max_ = 0.869
6874 measured reflections
6718 independent reflections
5170 reflections with *I* > 2σ(*I*)
*R*_int_ = 0.026
3 standard reflections every 3 reflections intensity decay: 4.0%

#### Refinement

*R*[*F* ^2^ > 2σ(*F* ^2^)] = 0.032
*wR*(*F* ^2^) = 0.082
*S* = 1.03
6718 reflections
370 parameters
H-atom parameters constrained
Δρ_max_ = 0.39 e Å^−3^
Δρ_min_ = −0.27 e Å^−3^

### 

Data collection: *CAD-4 EXPRESS* (Enraf–Nonius, 1994 ▶); cell refinement: *CAD-4 EXPRESS*; data reduction: *XCAD4* (Harms & Wocadlo, 1995 ▶); program(s) used to solve structure: *SIR2002* (Burla *et al.*, 2003 ▶); program(s) used to refine structure: *SHELXL97* (Sheldrick, 2008 ▶) and SQUEEZE in *PLATON* (Spek, 2009 ▶); molecular graphics: *ORTEP-3 for Windows* (Farrugia, 1997 ▶); software used to prepare material for publication: *WinGX* (Farrugia, 1999 ▶).

## Supplementary Material

Crystal structure: contains datablock(s) global, I. DOI: 10.1107/S1600536812043528/lh5542sup1.cif

Structure factors: contains datablock(s) I. DOI: 10.1107/S1600536812043528/lh5542Isup2.hkl

Additional supplementary materials:  crystallographic information; 3D view; checkCIF report

## Figures and Tables

**Fig. 1.**
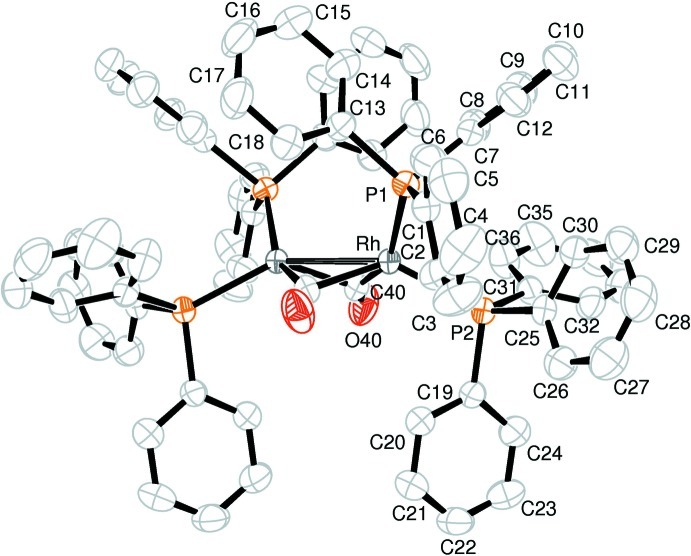
View of (I) (50% probability displacement ellipsoids). Unlabeled atoms are related by the symmetry operator (-x+2, y, -z+3/2).

## supplementary crystallographic information

### Comment

The dirhodium complex (I), (Rh(CO)(PPh_3_)_2_)_2_, is a precursor for HRh(CO)_2_(PPh_3_)_2_, a hydroformylation catalyst. A less precise crystal structure of I, as the dichloromethane solvate, was reported by Singh *et al.* (1973). In both solvates, the complex lies on a crystallographic twofold axis, with Rh—Rh = 2.6266 (8) Å (Singh *et al.*: 2.630 (1) Å). The four atoms proximate to Rh (Rh—P1 = 2.3222 (7), Rh—P2 = 2.3283 (8), Rh—C40 = 1.961 (3), Rh—C40' = 2.045 (3) Å) form a distorted tetrahedron, but the six angles deviate markedly from the ideal tetrahedral angle (δ_r.m.s._ = 23 (1)°). The angles approximate more closely those of a trigonal bipyramid (δ_r.m.s._ = 14 (1)°), with P1 and C40 in axial and P2 and C40' in equatorial positions, wth one equatorial position vacant. The two bridging carbonyl ligands do not lie in the same plane, the two Rh_2_CO planes forming a dihedral angle of 45.43 (5)°. Furthermore, each carbonyl is asymmetrically coordinated to the two Rh atoms, with a Rh—C distance of 1.961 (3) Å and Rh—C≡O angle 151.0 (2)° to one Rh, 2.045 (3) Å and 127.0 (2)° to the other. This asymmetric carbonyl bridging is also seen in the DCM solvate structure (Singh *et al.*, 1973).

### Experimental

Rh_2_(CO)_2_(PPh_3_)_4_ was synthesized from Rh(acac)(CO)_2_ and excess triphenylphosphine under hydroformylation conditions, with acetone as solvent: A small autoclave was charged with a solution of Rh(acac)(CO)_2_ (0.010 g), PPh_3_ (9.750 g), and 1-hexene 7.883 g) in acetone (40 ml) while inside a glovebox. The autoclave was sealed, removed from the glovebox, and placed in a heating mantle. The headspace of the autoclave was purged with *syn* gas (1:1 H_2_:CO, 3 *x* 60psig purges), and pressurized to 60psig with *syn* gas. The vessel was heated to 80°C and the *syn* gas pressure was adjusted to 80psig. After 18 h, the vessel was depressurized and purged with nitrogen (3x 60psig). The reaction mixture was analyzed with ^1^H NMR: 91% aldehydes, 6.7% olefin isomerization, 2.2% 1-hexene. The n/i ratio of the aldehydes were 13.6. The reaction mixture slowly concentrated upon sitting in a glove box to afford a few red crystals of (I).

### Refinement

Each cavity (estimated volume 288 Å^3^) associated with a dirhodium molecule contains two disordered acetone molecules, for which no reasonable model could be developed. Therefore, the observed structure amplitudes were modified by *PLATON*/SQUEEZE (Spek, 2009) to subtract the scattering contribution of the electron density found in each cavity.

All H atoms were placed in calculated positions, with C(*sp*^3^)—H = 0.96 Å, *U*_iso_(H) = 1.5*U*_eq_(C), and C(*sp*^2^)—H = 0.93 Å, *U*_iso_(H) = 1.2*U*_eq_(C), and thereafter allowed to ride the attached C atom.

### Crystal data

**Table:**

[Rh_2_(C_18_H_15_P)_4_(CO)_2_]·2C_3_H_6_O	*F*(000) = 2936
*M**_r_* = 1427.16	*D*_x_ = 1.386 Mg m^−^^3^
Monoclinic, *C*2/*c*	Mo *K*α radiation, λ = 0.71073 Å
Hall symbol: -C 2yc	Cell parameters from 25 reflections
*a* = 23.535 (3) Å	θ = 2.6–27.5°
*b* = 13.0758 (11) Å	µ = 0.63 mm^−^^1^
*c* = 24.650 (2) Å	*T* = 298 K
β = 115.67 (2)°	Prism, red
*V* = 6837.1 (16) Å^3^	0.38 × 0.38 × 0.23 mm
*Z* = 4	

### Data collection

**Table:**

Enraf–Nonius CAD-4 diffractometer	5170 reflections with *I* > 2σ(*I*)
Radiation source: fine-focus sealed tube	*R*_int_ = 0.026
Graphite monochromator	θ_max_ = 26.0°, θ_min_ = 1.8°
θ/2θ scans	*h* = 0→28
Absorption correction: ψ scan (North *et al.*, 1968)	*k* = 0→16
*T*_min_ = 0.797, *T*_max_ = 0.869	*l* = −30→27
6874 measured reflections	3 standard reflections every 3 reflections
6718 independent reflections	intensity decay: 4.0%

### Refinement

**Table:**

Refinement on *F*^2^	Primary atom site location: structure-invariant direct methods
Least-squares matrix: full	Secondary atom site location: difference Fourier map
*R*[*F*^2^ > 2σ(*F*^2^)] = 0.032	Hydrogen site location: inferred from neighbouring sites
*wR*(*F*^2^) = 0.082	H-atom parameters constrained
*S* = 1.03	*w* = 1/[σ^2^(*F*_o_^2^) + (0.0423*P*)^2^ + 0.3727*P*] where *P* = (*F*_o_^2^ + 2*F*_c_^2^)/3
6718 reflections	(Δ/σ)_max_ = 0.003
370 parameters	Δρ_max_ = 0.39 e Å^−^^3^
0 restraints	Δρ_min_ = −0.27 e Å^−^^3^
0 constraints	

### Special details

**Table:**

Geometry. All e.s.d.'s (except the e.s.d. in the dihedral angle between two l.s. planes) are estimated using the full covariance matrix. The cell e.s.d.'s are taken into account individually in the estimation of e.s.d.'s in distances, angles and torsion angles; correlations between e.s.d.'s in cell parameters are only used when they are defined by crystal symmetry. An approximate (isotropic) treatment of cell e.s.d.'s is used for estimating e.s.d.'s involving l.s. planes.

### Fractional atomic coordinates and isotropic or equivalent isotropic displacement parameters (Å^2^)

**Table:**

	*x*	*y*	*z*	*U*_iso_*/*U*_eq_	
P1	0.88187 (3)	−0.11053 (5)	0.72824 (3)	0.03726 (16)	
C1	0.81798 (13)	−0.0719 (2)	0.74770 (11)	0.0410 (6)	
C2	0.80127 (16)	0.0298 (2)	0.74235 (14)	0.0549 (8)	
H2	0.8229	0.0768	0.73	0.066*	
C3	0.7526 (2)	0.0623 (3)	0.75522 (17)	0.0752 (11)	
H3	0.7419	0.1313	0.7518	0.09*	
C4	0.71999 (19)	−0.0065 (3)	0.77297 (17)	0.0768 (11)	
H4	0.6865	0.0155	0.7804	0.092*	
C5	0.73669 (16)	−0.1068 (3)	0.77967 (14)	0.0621 (9)	
H5	0.7148	−0.1534	0.792	0.075*	
C6	0.78618 (15)	−0.1396 (2)	0.76812 (13)	0.0524 (7)	
H6	0.7983	−0.2079	0.7741	0.063*	
C7	0.84532 (13)	−0.19569 (19)	0.66282 (12)	0.0411 (6)	
C8	0.87874 (15)	−0.2110 (2)	0.62867 (14)	0.0547 (8)	
H8	0.9177	−0.1797	0.6398	0.066*	
C9	0.85363 (17)	−0.2730 (3)	0.57799 (15)	0.0671 (10)	
H9	0.8756	−0.2824	0.5548	0.081*	
C10	0.79721 (18)	−0.3200 (3)	0.56208 (15)	0.0706 (10)	
H10	0.7811	−0.3625	0.5285	0.085*	
C11	0.76397 (18)	−0.3051 (3)	0.59513 (16)	0.0694 (10)	
H11	0.7249	−0.3363	0.5835	0.083*	
C12	0.78835 (15)	−0.2437 (2)	0.64586 (13)	0.0560 (8)	
H12	0.7659	−0.2349	0.6686	0.067*	
C13	0.92309 (13)	−0.2033 (2)	0.78900 (12)	0.0436 (6)	
C14	0.93637 (16)	−0.3018 (2)	0.77782 (15)	0.0613 (9)	
H14	0.9245	−0.3234	0.7384	0.074*	
C15	0.96749 (18)	−0.3687 (3)	0.82533 (18)	0.0770 (11)	
H15	0.9769	−0.4345	0.8174	0.092*	
C16	0.98448 (17)	−0.3396 (3)	0.88318 (17)	0.0736 (11)	
H16	1.0041	−0.3856	0.9146	0.088*	
C17	0.97239 (17)	−0.2422 (3)	0.89466 (15)	0.0717 (10)	
H17	0.9847	−0.2213	0.9342	0.086*	
C18	0.94185 (15)	−0.1738 (3)	0.84792 (14)	0.0585 (8)	
H18	0.934	−0.1074	0.8564	0.07*	
P2	0.86831 (3)	0.09673 (5)	0.62360 (3)	0.03564 (16)	
C19	0.88124 (12)	0.2359 (2)	0.63031 (12)	0.0401 (6)	
C20	0.90876 (14)	0.2779 (2)	0.68686 (13)	0.0521 (8)	
H20	0.9236	0.2352	0.7203	0.063*	
C21	0.91480 (16)	0.3830 (2)	0.69503 (15)	0.0636 (9)	
H21	0.9334	0.4101	0.7337	0.076*	
C22	0.89343 (16)	0.4465 (2)	0.64633 (17)	0.0654 (9)	
H22	0.8971	0.517	0.6518	0.079*	
C23	0.86680 (18)	0.4065 (3)	0.58989 (17)	0.0717 (10)	
H23	0.8526	0.4499	0.5567	0.086*	
C24	0.86062 (16)	0.3016 (2)	0.58135 (14)	0.0593 (8)	
H24	0.8425	0.275	0.5425	0.071*	
C25	0.78347 (12)	0.0915 (2)	0.60350 (11)	0.0398 (6)	
C26	0.74864 (14)	0.1729 (2)	0.60884 (13)	0.0523 (7)	
H26	0.7679	0.2362	0.6214	0.063*	
C27	0.68550 (16)	0.1614 (3)	0.59578 (16)	0.0680 (9)	
H27	0.6628	0.2171	0.5995	0.082*	
C28	0.65644 (16)	0.0694 (3)	0.57752 (16)	0.0685 (10)	
H28	0.6143	0.0619	0.5697	0.082*	
C29	0.68964 (15)	−0.0129 (3)	0.57065 (15)	0.0597 (8)	
H29	0.6696	−0.0755	0.5574	0.072*	
C30	0.75280 (14)	−0.0021 (2)	0.58356 (13)	0.0470 (7)	
H30	0.775	−0.0577	0.5789	0.056*	
C31	0.86728 (13)	0.0638 (2)	0.55073 (12)	0.0414 (6)	
C32	0.81587 (15)	0.0855 (2)	0.49619 (12)	0.0545 (8)	
H32	0.7809	0.1187	0.4962	0.065*	
C33	0.81608 (19)	0.0587 (3)	0.44229 (14)	0.0676 (10)	
H33	0.7815	0.0745	0.4063	0.081*	
C34	0.8669 (2)	0.0088 (3)	0.44120 (16)	0.0728 (11)	
H34	0.8673	−0.0083	0.4047	0.087*	
C35	0.9171 (2)	−0.0156 (3)	0.49460 (19)	0.0835 (12)	
H35	0.9514	−0.0503	0.4942	0.1*	
C36	0.91725 (16)	0.0110 (3)	0.54926 (15)	0.0639 (9)	
H36	0.9513	−0.007	0.5851	0.077*	
Rh1	0.941218 (9)	0.013747 (14)	0.708963 (9)	0.03409 (7)	
C40	1.01769 (13)	0.0593 (2)	0.70298 (12)	0.0435 (7)	
O40	1.04318 (10)	0.09216 (19)	0.67507 (10)	0.0660 (6)	

### Atomic displacement parameters (Å^2^)

**Table:**

	*U*^11^	*U*^22^	*U*^33^	*U*^12^	*U*^13^	*U*^23^
P1	0.0398 (4)	0.0314 (3)	0.0343 (3)	−0.0018 (3)	0.0101 (3)	−0.0004 (3)
C1	0.0425 (15)	0.0420 (16)	0.0325 (13)	−0.0010 (12)	0.0106 (12)	−0.0010 (11)
C2	0.072 (2)	0.0469 (18)	0.0517 (18)	0.0104 (15)	0.0326 (16)	0.0081 (14)
C3	0.094 (3)	0.064 (2)	0.081 (3)	0.031 (2)	0.051 (2)	0.015 (2)
C4	0.075 (3)	0.101 (3)	0.071 (2)	0.027 (2)	0.048 (2)	0.016 (2)
C5	0.063 (2)	0.078 (2)	0.0519 (19)	−0.0085 (18)	0.0311 (17)	0.0064 (17)
C6	0.0587 (19)	0.0476 (17)	0.0506 (17)	−0.0074 (14)	0.0234 (15)	−0.0024 (14)
C7	0.0443 (15)	0.0309 (13)	0.0369 (14)	0.0001 (11)	0.0071 (12)	−0.0004 (11)
C8	0.0515 (18)	0.0554 (19)	0.0513 (18)	0.0051 (15)	0.0169 (15)	−0.0078 (14)
C9	0.074 (2)	0.068 (2)	0.0511 (19)	0.0114 (19)	0.0190 (17)	−0.0148 (17)
C10	0.080 (3)	0.056 (2)	0.0483 (19)	0.0008 (19)	0.0029 (18)	−0.0171 (16)
C11	0.072 (2)	0.059 (2)	0.063 (2)	−0.0199 (18)	0.0158 (19)	−0.0109 (17)
C12	0.061 (2)	0.0533 (19)	0.0499 (17)	−0.0183 (15)	0.0205 (15)	−0.0082 (14)
C13	0.0429 (15)	0.0394 (15)	0.0425 (15)	0.0003 (12)	0.0129 (13)	0.0071 (12)
C14	0.067 (2)	0.0473 (18)	0.0561 (19)	0.0123 (16)	0.0140 (16)	0.0040 (15)
C15	0.081 (3)	0.049 (2)	0.079 (3)	0.0132 (19)	0.014 (2)	0.0149 (19)
C16	0.063 (2)	0.067 (2)	0.073 (3)	0.0084 (18)	0.0131 (19)	0.035 (2)
C17	0.071 (2)	0.084 (3)	0.0462 (19)	0.000 (2)	0.0128 (17)	0.0166 (18)
C18	0.067 (2)	0.0502 (18)	0.0500 (18)	−0.0006 (16)	0.0172 (16)	0.0045 (14)
P2	0.0333 (3)	0.0350 (4)	0.0313 (3)	0.0018 (3)	0.0071 (3)	0.0008 (3)
C19	0.0349 (14)	0.0352 (14)	0.0426 (15)	0.0018 (11)	0.0097 (11)	0.0019 (11)
C20	0.0530 (18)	0.0422 (16)	0.0472 (17)	0.0042 (14)	0.0087 (14)	−0.0011 (13)
C21	0.067 (2)	0.0438 (18)	0.062 (2)	−0.0012 (16)	0.0106 (16)	−0.0124 (15)
C22	0.066 (2)	0.0346 (16)	0.087 (3)	−0.0006 (15)	0.025 (2)	−0.0032 (17)
C23	0.088 (3)	0.0410 (18)	0.075 (2)	0.0024 (17)	0.025 (2)	0.0164 (17)
C24	0.072 (2)	0.0459 (18)	0.0479 (18)	−0.0008 (16)	0.0146 (16)	0.0047 (14)
C25	0.0343 (14)	0.0432 (15)	0.0334 (13)	0.0033 (11)	0.0067 (11)	0.0032 (11)
C26	0.0454 (17)	0.0526 (18)	0.0557 (18)	0.0042 (14)	0.0188 (15)	−0.0013 (14)
C27	0.051 (2)	0.075 (2)	0.082 (2)	0.0142 (18)	0.0320 (19)	0.0062 (19)
C28	0.0387 (17)	0.086 (3)	0.076 (2)	−0.0043 (18)	0.0207 (17)	0.012 (2)
C29	0.0460 (17)	0.060 (2)	0.064 (2)	−0.0115 (15)	0.0156 (15)	0.0076 (16)
C30	0.0418 (15)	0.0487 (17)	0.0435 (15)	−0.0005 (13)	0.0118 (12)	0.0039 (12)
C31	0.0430 (15)	0.0404 (15)	0.0372 (14)	−0.0042 (12)	0.0141 (12)	−0.0014 (11)
C32	0.062 (2)	0.0548 (18)	0.0376 (15)	0.0089 (15)	0.0132 (14)	0.0042 (13)
C33	0.092 (3)	0.063 (2)	0.0361 (16)	−0.003 (2)	0.0164 (17)	0.0008 (15)
C34	0.096 (3)	0.081 (3)	0.051 (2)	−0.026 (2)	0.040 (2)	−0.0163 (18)
C35	0.071 (3)	0.111 (3)	0.080 (3)	−0.006 (2)	0.044 (2)	−0.027 (2)
C36	0.0499 (18)	0.085 (3)	0.0538 (19)	0.0008 (17)	0.0195 (15)	−0.0136 (17)
Rh1	0.03109 (11)	0.03049 (11)	0.03132 (11)	−0.00057 (8)	0.00472 (8)	0.00033 (8)
C40	0.0425 (16)	0.0366 (14)	0.0378 (14)	−0.0003 (12)	0.0047 (12)	0.0054 (12)
O40	0.0493 (13)	0.0884 (17)	0.0531 (13)	−0.0058 (12)	0.0154 (11)	0.0280 (12)

### Geometric parameters (Å, º)

**Table:**

P1—C1	1.836 (3)	P2—Rh1	2.3283 (8)
P1—C7	1.838 (3)	C19—C20	1.372 (4)
P1—C13	1.843 (3)	C19—C24	1.386 (4)
P1—Rh1	2.3222 (7)	C20—C21	1.387 (4)
C1—C2	1.376 (4)	C20—H20	0.93
C1—C6	1.387 (4)	C21—C22	1.364 (5)
C2—C3	1.382 (5)	C21—H21	0.93
C2—H2	0.93	C22—C23	1.359 (5)
C3—C4	1.372 (5)	C22—H22	0.93
C3—H3	0.93	C23—C24	1.386 (4)
C4—C5	1.359 (5)	C23—H23	0.93
C4—H4	0.93	C24—H24	0.93
C5—C6	1.382 (4)	C25—C26	1.384 (4)
C5—H5	0.93	C25—C30	1.397 (4)
C6—H6	0.93	C26—C27	1.386 (4)
C7—C12	1.372 (4)	C26—H26	0.93
C7—C8	1.393 (4)	C27—C28	1.361 (5)
C8—C9	1.389 (4)	C27—H27	0.93
C8—H8	0.93	C28—C29	1.383 (5)
C9—C10	1.358 (5)	C28—H28	0.93
C9—H9	0.93	C29—C30	1.386 (4)
C10—C11	1.366 (5)	C29—H29	0.93
C10—H10	0.93	C30—H30	0.93
C11—C12	1.384 (4)	C31—C36	1.377 (4)
C11—H11	0.93	C31—C32	1.394 (4)
C12—H12	0.93	C32—C33	1.376 (4)
C13—C18	1.378 (4)	C32—H32	0.93
C13—C14	1.381 (4)	C33—C34	1.373 (5)
C14—C15	1.389 (4)	C33—H33	0.93
C14—H14	0.93	C34—C35	1.372 (6)
C15—C16	1.358 (5)	C34—H34	0.93
C15—H15	0.93	C35—C36	1.390 (5)
C16—C17	1.361 (5)	C35—H35	0.93
C16—H16	0.93	C36—H36	0.93
C17—C18	1.388 (4)	Rh1—C40	1.961 (3)
C17—H17	0.93	Rh1—C40^i^	2.045 (3)
C18—H18	0.93	Rh1—Rh1^i^	2.6266 (8)
P2—C31	1.837 (3)	C40—O40	1.173 (3)
P2—C25	1.838 (3)	C40—Rh1^i^	2.045 (3)
P2—C19	1.841 (3)		
			
C1—P1—C7	105.72 (13)	C20—C19—P2	118.3 (2)
C1—P1—C13	99.86 (13)	C24—C19—P2	123.5 (2)
C7—P1—C13	101.28 (12)	C19—C20—C21	121.2 (3)
C1—P1—Rh1	119.62 (9)	C19—C20—H20	119.4
C7—P1—Rh1	109.80 (9)	C21—C20—H20	119.4
C13—P1—Rh1	118.38 (9)	C22—C21—C20	120.0 (3)
C2—C1—C6	118.3 (3)	C22—C21—H21	120
C2—C1—P1	118.2 (2)	C20—C21—H21	120
C6—C1—P1	123.4 (2)	C23—C22—C21	119.8 (3)
C1—C2—C3	120.4 (3)	C23—C22—H22	120.1
C1—C2—H2	119.8	C21—C22—H22	120.1
C3—C2—H2	119.8	C22—C23—C24	120.5 (3)
C4—C3—C2	120.3 (3)	C22—C23—H23	119.7
C4—C3—H3	119.8	C24—C23—H23	119.7
C2—C3—H3	119.8	C19—C24—C23	120.4 (3)
C5—C4—C3	120.0 (3)	C19—C24—H24	119.8
C5—C4—H4	120	C23—C24—H24	119.8
C3—C4—H4	120	C26—C25—C30	118.0 (3)
C4—C5—C6	120.0 (3)	C26—C25—P2	124.4 (2)
C4—C5—H5	120	C30—C25—P2	117.6 (2)
C6—C5—H5	120	C25—C26—C27	121.0 (3)
C5—C6—C1	120.8 (3)	C25—C26—H26	119.5
C5—C6—H6	119.6	C27—C26—H26	119.5
C1—C6—H6	119.6	C28—C27—C26	120.5 (3)
C12—C7—C8	118.9 (3)	C28—C27—H27	119.8
C12—C7—P1	124.7 (2)	C26—C27—H27	119.8
C8—C7—P1	116.4 (2)	C27—C28—C29	119.9 (3)
C9—C8—C7	119.7 (3)	C27—C28—H28	120
C9—C8—H8	120.1	C29—C28—H28	120
C7—C8—H8	120.1	C28—C29—C30	119.9 (3)
C10—C9—C8	120.4 (3)	C28—C29—H29	120
C10—C9—H9	119.8	C30—C29—H29	120
C8—C9—H9	119.8	C29—C30—C25	120.6 (3)
C9—C10—C11	120.3 (3)	C29—C30—H30	119.7
C9—C10—H10	119.9	C25—C30—H30	119.7
C11—C10—H10	119.9	C36—C31—C32	118.1 (3)
C10—C11—C12	120.1 (3)	C36—C31—P2	119.6 (2)
C10—C11—H11	120	C32—C31—P2	122.2 (2)
C12—C11—H11	120	C33—C32—C31	121.0 (3)
C7—C12—C11	120.6 (3)	C33—C32—H32	119.5
C7—C12—H12	119.7	C31—C32—H32	119.5
C11—C12—H12	119.7	C34—C33—C32	120.5 (3)
C18—C13—C14	118.4 (3)	C34—C33—H33	119.7
C18—C13—P1	119.2 (2)	C32—C33—H33	119.7
C14—C13—P1	122.5 (2)	C35—C34—C33	119.1 (3)
C13—C14—C15	120.1 (3)	C35—C34—H34	120.4
C13—C14—H14	120	C33—C34—H34	120.4
C15—C14—H14	120	C34—C35—C36	120.8 (4)
C16—C15—C14	121.1 (3)	C34—C35—H35	119.6
C16—C15—H15	119.4	C36—C35—H35	119.6
C14—C15—H15	119.4	C31—C36—C35	120.4 (3)
C15—C16—C17	119.2 (3)	C31—C36—H36	119.8
C15—C16—H16	120.4	C35—C36—H36	119.8
C17—C16—H16	120.4	C40—Rh1—C40^i^	87.93 (14)
C16—C17—C18	120.7 (3)	C40—Rh1—P1	151.76 (8)
C16—C17—H17	119.7	C40^i^—Rh1—P1	92.09 (9)
C18—C17—H17	119.7	C40—Rh1—P2	97.56 (8)
C13—C18—C17	120.6 (3)	C40^i^—Rh1—P2	130.38 (8)
C13—C18—H18	119.7	P1—Rh1—P2	103.76 (3)
C17—C18—H18	119.7	C40—Rh1—Rh1^i^	50.44 (8)
C31—P2—C25	100.08 (12)	C40^i^—Rh1—Rh1^i^	47.66 (8)
C31—P2—C19	104.56 (13)	P1—Rh1—Rh1^i^	111.635 (19)
C25—P2—C19	100.32 (12)	P2—Rh1—Rh1^i^	144.50 (2)
C31—P2—Rh1	117.82 (9)	O40—C40—Rh1	151.0 (2)
C25—P2—Rh1	121.02 (9)	O40—C40—Rh1^i^	127.0 (2)
C19—P2—Rh1	110.55 (8)	Rh1—C40—Rh1^i^	81.90 (11)
C20—C19—C24	118.0 (3)		
			
C7—P1—C1—C2	−115.7 (2)	Rh1—P2—C25—C26	107.8 (2)
C13—P1—C1—C2	139.5 (2)	C31—P2—C25—C30	61.5 (2)
Rh1—P1—C1—C2	8.7 (3)	C19—P2—C25—C30	168.5 (2)
C7—P1—C1—C6	65.1 (3)	Rh1—P2—C25—C30	−69.8 (2)
C13—P1—C1—C6	−39.7 (3)	C30—C25—C26—C27	1.1 (4)
Rh1—P1—C1—C6	−170.5 (2)	P2—C25—C26—C27	−176.5 (2)
C6—C1—C2—C3	−2.1 (5)	C25—C26—C27—C28	0.2 (5)
P1—C1—C2—C3	178.6 (3)	C26—C27—C28—C29	−1.4 (6)
C1—C2—C3—C4	−0.6 (6)	C27—C28—C29—C30	1.3 (5)
C2—C3—C4—C5	1.9 (6)	C28—C29—C30—C25	0.0 (5)
C3—C4—C5—C6	−0.5 (6)	C26—C25—C30—C29	−1.1 (4)
C4—C5—C6—C1	−2.2 (5)	P2—C25—C30—C29	176.6 (2)
C2—C1—C6—C5	3.5 (4)	C25—P2—C31—C36	−148.2 (3)
P1—C1—C6—C5	−177.3 (2)	C19—P2—C31—C36	108.3 (3)
C1—P1—C7—C12	−18.3 (3)	Rh1—P2—C31—C36	−14.9 (3)
C13—P1—C7—C12	85.5 (3)	C25—P2—C31—C32	28.3 (3)
Rh1—P1—C7—C12	−148.6 (2)	C19—P2—C31—C32	−75.2 (3)
C1—P1—C7—C8	161.5 (2)	Rh1—P2—C31—C32	161.6 (2)
C13—P1—C7—C8	−94.8 (2)	C36—C31—C32—C33	−2.6 (5)
Rh1—P1—C7—C8	31.2 (2)	P2—C31—C32—C33	−179.1 (3)
C12—C7—C8—C9	0.8 (4)	C31—C32—C33—C34	0.7 (5)
P1—C7—C8—C9	−179.0 (2)	C32—C33—C34—C35	1.1 (6)
C7—C8—C9—C10	−1.0 (5)	C33—C34—C35—C36	−1.0 (6)
C8—C9—C10—C11	1.3 (6)	C32—C31—C36—C35	2.7 (5)
C9—C10—C11—C12	−1.4 (6)	P2—C31—C36—C35	179.3 (3)
C8—C7—C12—C11	−1.0 (5)	C34—C35—C36—C31	−1.0 (6)
P1—C7—C12—C11	178.8 (2)	C1—P1—Rh1—C40	158.53 (19)
C10—C11—C12—C7	1.3 (5)	C7—P1—Rh1—C40	−79.1 (2)
C1—P1—C13—C18	−53.5 (3)	C13—P1—Rh1—C40	36.5 (2)
C7—P1—C13—C18	−161.9 (3)	C1—P1—Rh1—C40^i^	69.02 (13)
Rh1—P1—C13—C18	78.1 (3)	C7—P1—Rh1—C40^i^	−168.58 (12)
C1—P1—C13—C14	126.8 (3)	C13—P1—Rh1—C40^i^	−53.06 (13)
C7—P1—C13—C14	18.5 (3)	C1—P1—Rh1—P2	−63.55 (10)
Rh1—P1—C13—C14	−101.5 (3)	C7—P1—Rh1—P2	58.85 (9)
C18—C13—C14—C15	0.5 (5)	C13—P1—Rh1—P2	174.37 (11)
P1—C13—C14—C15	−179.9 (3)	C1—P1—Rh1—Rh1^i^	113.74 (9)
C13—C14—C15—C16	1.1 (6)	C7—P1—Rh1—Rh1^i^	−123.86 (9)
C14—C15—C16—C17	−2.0 (6)	C13—P1—Rh1—Rh1^i^	−8.34 (11)
C15—C16—C17—C18	1.4 (6)	C31—P2—Rh1—C40	59.88 (13)
C14—C13—C18—C17	−1.1 (5)	C25—P2—Rh1—C40	−176.87 (13)
P1—C13—C18—C17	179.3 (3)	C19—P2—Rh1—C40	−60.21 (13)
C16—C17—C18—C13	0.2 (5)	C31—P2—Rh1—C40^i^	153.61 (15)
C31—P2—C19—C20	−154.4 (2)	C25—P2—Rh1—C40^i^	−83.14 (15)
C25—P2—C19—C20	102.2 (2)	C19—P2—Rh1—C40^i^	33.52 (15)
Rh1—P2—C19—C20	−26.6 (3)	C31—P2—Rh1—P1	−101.46 (10)
C31—P2—C19—C24	30.4 (3)	C25—P2—Rh1—P1	21.78 (11)
C25—P2—C19—C24	−73.0 (3)	C19—P2—Rh1—P1	138.45 (10)
Rh1—P2—C19—C24	158.1 (2)	C31—P2—Rh1—Rh1^i^	82.89 (11)
C24—C19—C20—C21	1.2 (5)	C25—P2—Rh1—Rh1^i^	−153.87 (10)
P2—C19—C20—C21	−174.3 (3)	C19—P2—Rh1—Rh1^i^	−37.21 (11)
C19—C20—C21—C22	−0.3 (5)	C40^i^—Rh1—C40—O40	−150.3 (5)
C20—C21—C22—C23	−0.6 (6)	P1—Rh1—C40—O40	119.1 (5)
C21—C22—C23—C24	0.6 (6)	P2—Rh1—C40—O40	−19.8 (5)
C20—C19—C24—C23	−1.2 (5)	Rh1^i^—Rh1—C40—O40	177.3 (6)
P2—C19—C24—C23	174.0 (3)	C40^i^—Rh1—C40—Rh1^i^	32.40 (14)
C22—C23—C24—C19	0.3 (6)	P1—Rh1—C40—Rh1^i^	−58.2 (2)
C31—P2—C25—C26	−120.9 (3)	P2—Rh1—C40—Rh1^i^	162.88 (6)
C19—P2—C25—C26	−13.9 (3)		

Symmetry code: (i) −*x*+2, *y*, −*z*+3/2.
